# Resveratrol Attenuates Degeneration and Apoptosis of Cardiomyocytes Induced by Aortic Clamping

**DOI:** 10.21470/1678-9741-2023-0224

**Published:** 2023-09-19

**Authors:** Saban Ergene, Dogus Hemsinli, Sedat Ozan Karakisi, Levent Tümkaya, Tolga Mercantepe, Adnan Yilmaz, Ibrahim Yel

**Affiliations:** 1 Department of Cardiovascular Surgery, Faculty of Medicine, Recep Tayyip Erdogan University, Rize, Turkey; 2 Department of Histology and Embryology, Faculty of Medicine, Recep Tayyip Erdogan University, Rize, Turkey; 3 Department of Medical Biochemistry, Faculty of Medicine, Recep Tayyip Erdogan University, Rize, Turkey

**Keywords:** Abdominal Aorta, Glutathione Peroxidase, Myocardium, Malondialdehyde, Resveratrol

## Abstract

**Introduction:**

Objective: To investigate the potential beneficial effects of resveratrol (RVT) against ischemia-reperfusion injury of myocardial tissue during surgical treatment of ruptured abdominal aortic aneurysm.

**Methods:**

Four groups were established — control, ischemia/reperfusion (I/R), sham (I/R+solvent/dimethyl sulfoxide [^DMSO^]), and I/R+RVT. Ruptured abdominal aortic aneurysm model was used as the experimental protocol.

**Results:**

In the I/R and I/R+DMSO groups, malondialdehyde (MDA) levels in myocardial tissue were found to be significantly increased compared to the control group. The MDA level in myocardial tissue was significantly decreased in the I/ R+RVT group compared to the I/R group. In I/R and I/R+DMSO groups, glutathione peroxidase (GSH) levels in myocardial tissue were found to be significantly decreased compared to the control group. The GSH level in the myocardial tissue was significantly increased in the I/R+RVT group compared to the I/R group. In the light microscope, isotropic and anisotropic band disorganized atypical cardiomyocytes in the I/R group and degenerative cardiomyocytes and edematous areas in the I/R+DMSO group were observed. Degenerative cardiomyocytes and edematous areas were decreased in the I/R+RVT group. When heart tissue sections incubated with cleaved caspase-3 primary antibodies were examined under the light microscope, apoptotic cardiomyocytes were present in I/R and I/R+DMSO groups. A decrease in the number of apoptotic cardiomyocytes was observed in the I/R+RVT group.

**Conclusion:**

The findings of the present study indicate that RVT exhibits protective effects against ischemia-reperfusion injury occurring in the myocardium as a distant organ as a result of abdominal aorta clamping.

## INTRODUCTION

The development of endovascular repair techniques has made it possible to treat ruptured abdominal aortic aneurysms (rAAA) without open surgery and cross-clamping. Unfortunately, however, a significant proportion of rAAAs cannot be treated with endovascular approach due to inappropriate anatomical features and economic and logistical difficulties in obtaining stent grafts. No marked improvement has still been achieved in mortality rates in rAAA, which remains an important cause of mortality in men over 65 years old^[1]^. Also considering those patients who die with correct diagnoses before reaching the cardiovascular surgery team, rAAA is a much more important health problem than currently believed^[2]^. In rAAA, hypovolemic shock, which usually develops due to bleeding from the retroperitoneum, and sometimes into the abdomen, causes widespread perfusion disturbance in the entire body. Patients who reach the surgery team late are exposed to prolonged hypovolemic shock, resulting in acid-base balance disturbance and hypoperfusion findings in very distant organs. Although cross-clamping of the abdominal aorta and fluid replacement establish hemodynamic stability, the ischemic process continues in the trunk’s lower half until surgical repair is completed. Ischemia-reperfusion injury (IRI) developing during rAAA repair is a combination of this consecutive ischemic process and reperfusion^[1]^. The essential mechanisms of IRI are free oxygen radical (FOR) formation, a decrease in antioxidant enzymes, lipid peroxidation, neutrophil activation, and caspase-3-induced apoptosis. Myocardial injury caused by IRI is one of the main causes of death in the early postoperative period in patients undergoing rAAA surgery^[2^,^3]^. It has been suggested that a significant proportion of problems emerging during intensive care follow-up immediately after surgery develop due to myocardial dysfunction and that myocardial functions, therefore, need to be preserved for recovery^[3]^. Despite all the information currently available, the mechanism involved in myocardial IRI is still unclear, and there are numerous unknown interactions between the existing mechanisms^[4]^.

Resveratrol (RVT) is a natural polyphenol abundantly present in black grape seed. Studies have shown that, due to its powerful antioxidant and anti-inflammatory properties, it exhibits therapeutic effects on cancer, diabetes mellitus, and cardiovascular diseases^[2,4]^. This study aimed to investigate the potential beneficial effects of RVT against IRI development during rAAA formation and surgical treatment.

## METHODS

This study was designed and performed with the approval of the Recep Tayyip Erdogan University’s animal experiments ethical committee (approval number 2018-11). The research was conducted with 4-5-month male rats with a mean weight of 229±47 g. Four groups were established — control, ischemia/ reperfusion (I/R), sham (I/R+solvent/dimethyl sulfoxide [^DMSO^]), and I/R+RVT. Eight rats were randomly assigned to each group. Rats were fed with standard rat chow and tap water in polyethylene containers in our experimental animal application center and were cared for following the criteria set out in the Guide for the Care and Use of Laboratory Animals.

### Experimental Protocol

The rAAA model first described by Lindsay et al.^[5]^ and employed in our previous studies was used in the present research^[5,6,7]^. No procedure was performed on the rats in the control group apart from aortic exploration with median laparotomy. The rats in other groups were first exposed to hypovolemic shock to simulate hypovolemic shock in patients with rAAA. Hemodynamic stabilization was next performed through the application of a cross-clamp to the abdominal aorta. Ischemia was then applied for 60 minutes to simulate the surgical repair stage. Finally, reperfusion was applied for 120 minutes to simulate the stage when surgical repair is completed with cross-clamp removal.

The rats were anesthetized with 50 mg/kg ketamine hydrochloride (Ketalar®, Parke-Davis, Eczacibasi, Istanbul, Turkey) and 10 mg/ kg xylazine hydrochloride (Alfazyne®, Alfasan International B.V. Woerden, the Netherlands) administered via intraperitoneal route. The movement was thus prevented, while spontaneous respiration was preserved. After anesthetization, the rats were fixed supine under a heating lamp. The right internal jugular vein was located through an incision to the right side of the neck, and this was then cannulated for fluid replacement. Next, 3 ml/ kg/h 0.9% NaCl solution was administered through this to replace insensible fluid loss. The left common carotid artery was located through an incision to the left side of the neck and cannulated to observe mean arterial pressure (MAP).

To expose the I/R, sham, and I/R+RVT groups to hypovolemic shock, blood was drawn in a controlled manner through the cannula on the carotid artery and placed into an injector containing 500 IU heparin (Nevparin, 5000 U/mL), and MAP ≤ 50 mmHg was established for 60 minutes. The blood placed into the injector was prevented from clotting by the heparin and was kept at room temperature. On completion of the hypovolemic shock stimulation stage, a midline incision was performed on all rats under appropriate antiseptic conditions. Anesthesia was applied to the rats in the control group, with no additional procedure being performed, with heat and insensible fluid loss being prevented, and the abdominal incisions were closed with single sutures after four hours.

In the groups other than the control group, the abdominal aorta was located by the surgical opening of the retroperitoneum, and systemic heparinization was performed with 250 IU intravenous heparin. Two vascular clamps were then attached to the abdominal aorta immediately distal to the renal arteries and proximal to the iliac bifurcation. The blood kept at room temperature was returned through the venous cannula during the 60-minute ischemia stage in which surgical repair was simulated. On completion of the ischemia stage with the removal of the vascular clamps, the abdominal incision was closed with single sutures. During the subsequent 120-minute reperfusion stage, MAP was maintained at approximately 100 mmHg through appropriate fluid replacement^[5,7]^. In the I/R+RVT group, 10 mg/kg RVT was administered 15 minutes before the ischemia stage and 10 minutes before the reperfusion stage via the intraperitoneal route^[6,8]^. Rats in the sham group received an equal amount of intraperitoneal DMSO, and those in the I/R group an equal quantity of isotonic solution, intraperitoneally. At the end of the study, all rats were sacrificed by exsanguination through the cannulas on the carotid arteries.

### Biochemical Analysis

After cold phosphate buffer washing, cold phosphate buffer was added to the myocardial tissue specimens at a volume twice the tissue weight. All specimens were homogenized for one minute at 30 Hertz. The homogenized tissues were then centrifuged at 3000 g for 15 minutes at +4 °C, and the resulting supernatant was then subjected to biochemical analysis^[8]^.

### Tissue Malondialdehyde and Glutathione Level Measurement

Malondialdehyde (MDA) levels were measured using the method described by Draper and Hadley. This relies on MDA, the final product of lipid peroxidation, producing a pink complex by reacting with thiobarbituric acid (TBA) and yielding maximum absorbance at 532 nm^[9]^.

Glutathione (GSH) levels in myocardial tissues were measured using the Ellman method. The principle of this method relies on the spectrophotometric measurement of the color produced by the free sulfhydryl groups in the myocardial homogenate with Ellman’s reagent^[10]^.

### Histopathological Analysis

Myocardial tissues obtained from the rats were trimmed and fixed for 48 hours in 10% formalin (Sigma Aldrich, St. Louis, Missouri, United States of America). Following routine procedures, the samples were embedded in paraffin (Merck, Darmstadt, Germany). Next, sections 4-5 µm in thickness were taken using a microtome (Leica, RM2125RT, Germany) and stained with hematoxylin (Harris hematoxylin, Merck, Germany) and eosin (Eosin G, Merck, Germany) (hematoxylin and eosin [^H&E^]) and Goldner’s Masson trichrome (Merck, Darmstadt, Germany). Once the staining was complete, the tissue samples were examined under a light microscope (Olympus BX51, Olympus Corporation, Tokyo, Japan) and photographed with an Olympus DP71 camera (Olympus Corporation, Tokyo, Japan).

### Immunohistochemical Analysis

Avidin-biotin-peroxidase was employed to determine apoptotic cells in myocardial tissue. Sections 2-3 µm in thickness taken from the myocardial tissue paraffin blocks were placed onto positively charged slides. These sections were subsequently deparaffinized by being stored for 15 minutes in 3% H₂O₂ solution. A blocking solution was then applied for 20 seconds, after which the sections were incubated first with primary antibody (Caspase-3, Rabbit polyclonal, Abcam, United Kingdom) and then with secondary antibody (Goat Anti-Rabbit IgG H&L [^HRP^]) (ab205718, Abcam, United Kingdom) for 60 minutes. After being kept in diaminobenzidine chromogen (DAB Chromogen, Abcam, United Kingdom) solution for 15 minutes, the tissues were then counterstained with Harris hematoxylin (Merck, Darmstadt, Germany) and covered with an appropriate solution.

### Semi-Quantitative Analysis

To calculate heart pathological damage score (HPDS) values, the H&E-stained myocardial tissue sections were examined by two blinded histopathologists under the headings of disorganization of isotropic and anisotropic bands, degenerative cardiomyocytes, and edematous areas, as shown in Table 1. Analysis was performed on 45 randomly selected areas (× 40 objectives) in each myocardial tissue section.

**Table 1:** Heart pathological damage score.

Findings	Score
Disorganization of isotropic and anisotropic bands	0 (≤ 5%)
	1 (≤ 25%)
	2 (≤ 50%)
	3 (≤ 75%)
Degenerative cardiomyocytes	0 (≤ 5%)
	1 (≤ 25%)
	2 (≤ 50%)
	3 (≤ 75%)
Edema	0 (≤ 5%)
	1 (≤ 25%)
	2 (≤ 50%)
	3 (≤ 75%)

Heart tissue sections exposed to caspase-3 antibody were evaluated by two histopathologists blinded to the study for the calculation of caspase-3 primary antibody immune positivity score (Table 2). Scoring was performed on randomly selected 45 different areas in each objective (× 45).

**Table 2:** Caspase-3 primary antibody immune positivity scores.

Score	
0	None (< 5%)
1	Mild (< 25%)
2	Moderate (< 50%)
3	Severe (> 75%)

### Statistical Analysis

Data yielded by semi-quantitative and biochemical analyses were analyzed on SPSS Inc. Released 2009, PASW Statistics for Windows, version 18.00, Chicago: SPSS Inc. statistical software. Non-parametric data were calculated as the median and interquartile range (25%-75%) (maximum, minimum), while parametric data were calculated as mean plus standard deviation. Differences between the groups were analyzed using the Kruskal-Wallis and Tamhane’s T2 tests for non-parametric data, while parametric data were compared using one-way analysis of variance and Tukey’s honestly significant difference test. *P*-values < 0.05 were regarded as statistically significant.

## RESULTS

### Biochemical Analysis

A significant increase in MDA levels was observed in myocardial tissue from the I/R and I/R+DMSO groups compared with the control group (Table 3; *P*=0.000 and *P*=0.002, respectively). A significant decrease was determined in myocardial tissue MDA levels in the I/R+RVT group compared to the I/R group (Table 3; *P*=0.000). Additionally, a significant decrease in myocardial tissue GSH levels was observed in the I/R and I/R+DMSO groups compared to the control group (Table 3; *P*=0.006 and *P*=0.027, respectively). A significant increase in GSH levels in myocardial tissue was determined in the I/R+RVT group compared to the I/R group (Table 3; *P*=0.005).

**Table 3:** Biochemical results (mean ± standard deviation).

Groups	MDA (µmol/g tissue)	GSH (µmol/g tissue)
Control	15.49±1.18	4.77±0.92
I/R	23.02±3.28^a^	2.65±0.91^d^
I/R + DMSO	21.93±3.75^b^	3.04±1.01^e^
I/R + RVT	14.88±1.49^c^	4.79±1.05^f^

DMSO=dimethyl sulfoxide; GSH=glutathione; I/R=ischemia/reperfusion; MDA=malondialdehyde; RVT=resveratrol

a *P*=0.000 *vs.* the control group

b *P*=0.002 *vs.* the control group

c *P*=0.000 *vs.* the I/R group

d *P*=0.006 *vs.* the control group

e *P*=0.027 *vs.* the control group

f *P*=0.005 *vs.* the I/R group

One-way analysis of variance/Tukey’s honestly significant difference

### Histopathological Analysis

**Fig. 1:**
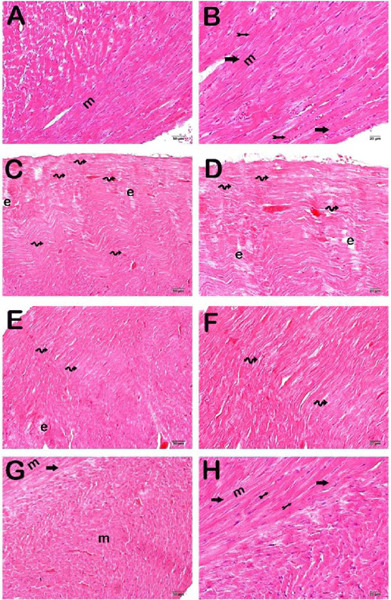
Light microscope photographs of myocardial tissue stained with hematoxylin and eosin. A (×20)-B (×40) – control group: normally organized isotropic and anisotropic bands (arrow) and discus intercalaris (tailed arrow) of cardiomyocytes (m) are observed (heart pathological damage score [^HPDS^] median:0[^0-1^]). C (×20)-D(×40) – ischemia/reperfusion (I/R) group: degenerative cardiomyocytes (spiral arrow) and edematous areas (e) with disorganized isotropic and anisotropic bands are observed (HPDS median: 5[^7-5^]). E (×20)-F (×40) – I/R+dimethyl sulfoxide group: degenerative cardiomyocytes and large edematous areas are commonly observed (HPDS median: 7[^7-7^]). G (×20)-H (×40) I/R+resveratrol group: a decrease in degenerative cardiomyocytes and large edematous areas and typical cardiomyocytes are observed (HPDS median:1[^1-2^]).

Light microscopic examination of heart tissue specimens from the control group revealed cardiomyocytes with normal isotropic and anisotropic bands (Figures 1 A-B; Table 4) (HPDS median: 1[^0-1^]). In contrast, atypical cardiomyocytes with impaired isotropic and anisotropic band organization were observed in the I/R group. Diffuse edematous areas were also present (Figures 1 C-D; Table 4) (HPDS median: 7[^5-7^]). Similarly, we observed degenerative cardiomyocytes and edematous areas in the I/ R+DMSO group (Figures 1 E-F; Table 4) (HPDS median: 7[^7-7^]). In contrast, we found fewer degenerative cardiomyocytes and edematous areas in the RVT treatment group (Figures 1 G-H; Table 4) (HPDS median: 1[^1-2^]).

**Table 4:** Semi-quantitative analysis results (median [^25%-75% interquartile range^]).

Group	Disorganization of isotropic and anisotropic bands	Degenerative cardiomyocytes	Edema	Heart pathological damage score
Control	0 (0-0)	0 (0-1)	0 (0-0)	1 (0-1)
I/R	3 (2-3)^a^	2.5 (2-3)^c^	1 (1-2)^e^	7 (5-7)^a^
I/R + DMSO	3 (2-3)^a^	3 (2-3)^c^	1 (1-2)^e^	7 (7-7)^a^
I/R + RVT	0.5 (0-1)^b^	0.5 (0-1)^d^	0 (0-1)^f^	1 (1-2)^b^

DMSO=dimethyl sulfoxide; I/R=ischemia/reperfusion; RVT=resveratrol

a *P*=0.000 *vs.* the control group

b *P*=0.000 *vs.* the I/R group

c *P*=0.001 *vs.* the control group

d *P*=0.001 *vs.* the I/R group

e *P*=0.01 *vs.* the control group

f *P*=0.01 *vs.* the I/R group

Kruskal-Wallis/Tamhane’s T2 test

### Semi-Quantitative Analysis

**Fig. 2:**
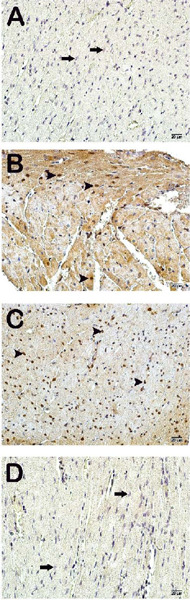
Light microscope images of heart tissue incubated with caspase-3 primary antibodies. A (×40) – control group: cardiomyocytes with the normal structure are observed in heart tissue sections (arrow) (caspase-3 primary antibody immune positivity score [^CPS^] median: 1[^0-1^]). B (×40) – ischemia/reperfusion (I/R) group: diffuse apoptotic cardiomyocytes are observed (arrowhead) (CPS median: 2[^2-3^]). C (×40) I/R+dimethyl sulfoxide group – widespread caspase-3 positivity is observed in cardiomyocytes (arrowhead) (CPS median: 2[^2-3^]). D (×40) I/R+resveratrol group: although a decrease in caspase-3 positivity is observed in cardiomyocytes, typical cardiomyocytes are observed (CPS median: 0.5[^0-1^]).

Scores for isotropic and anisotropic band disorganization, degenerative cardio myofibrils, edematous areas, and HPDS increased significantly in the I/R and I/R+DMSO groups compared to the control group (Figures 1 and 2; Table 4) (*P*=0.000, *P*=0.001, *P*=0.01, and *P*=0.000, respectively). Additionally, scores for isotropic and anisotropic band disorganization, degenerative cardiomyofibrils, edematous areas, and HPDS were significantly lower in the I/R+RVT group compared to the I/R group (Figures 1 and 2; Table 4) (*P*=0.000, *P*=0.001, *P*=0.01, and *P*=0.000, respectively).

### Immunohistochemical Analysis

Light microscopic examination of heart tissue sections incubated with cleaved caspase-3 primary antibody from the control group revealed normal cardiomyocytes (Figure 2 A; Table 5) (HPDS median: 1[^0-1^]). In contrast, apoptotic cardiomyocytes were present in the I/R and I/R+DMSO groups (Figure 2 B-C; Table 5) (HPDS median: 2[^2-3^]). However, a decrease in the number of apoptotic cardiomyocytes was determined in the I/R+RVT group (Figure 2 C; Table 5) (HPDS median: 0.5[^0-1^]).

**Table 5:** Immunohistochemical analysis results (median [^25%-75% interquartile range^]).

Group	CPS
Control	1 (0-1)
I/R	2 (2-3)^a^
I/R + DMSO	2 (2-3)^a^
I/R + RVT	0.5 (0-1)^b^

CPS=caspase-3 primary antibody immune positivity score; DMSO=dimethyl sulfoxide; I/R=ischemia/reperfusion; RVT=resveratrol

a *P*=0.000 *vs.* the control group

b *P*=0.001 *vs.* the I/R group

Kruskal-Wallis/Tamhane’s T2 test

## DISCUSSION

Despite significant improvements in emergency department procedures, surgical methods, and perioperative intensive care conditions, it has still not been possible to reduce rAAA mortality rates to under 50%^[11,12]^. The development of myocardial dysfunction plays a key role in the process leading to diffuse perfusion disorder and multiple organ failure, resulting in mortality in rAAA. Studies have shown that several factors contribute to the development of myocardial IRI in rAAA patients^[4]^. The hypovolemic shock caused by the rupture and aortic clamping has been reported to reduce myocardial functions by 65%^[1]^. Myocardial IRI is thought to be the main destructive mechanism of the inflammatory response responsible for the release of FOR and cytotoxic agents, resulting in cell damage^[4,13,14]^. Myocardial damage becomes particularly evident when the increase in the number of FOR exceeds the detoxification capacity of the antioxidant defense mechanism in the cardiac tissue, especially during the acute reperfusion period when aortic clamps are removed^[13]^. Endothelial damage, increased vascular permeability, tissue edema, and obstructions in small blood vessels occur under the effect of the released FOR and inflammatory mediators^[14]^.

The majority of studies examining IRI in myocardial tissue have been designed using the left anterior descending artery (LAD) occlusion model and have focused on isolated reperfusion injury in myocardial tissue. Very few studies have examined IRI developing in the myocardium as a distant organ as a result of abdominal aortic clamping. One such study determined deterioration in the structure of myocardial tissue, breaks in myofibrils, and swelling as a result of IRI induced by aortic clamping^[3]^. Studies involving LAD occlusion have observed structural deterioration in myocardial tissue, cell swelling, and focal necrosis, neutrophil infiltration in the interstitial area, vacuolar degeneration in cardiomyocytes, and significant mitochondrial swelling as a result of IRI^[15,16]^. Similarly, in the present study, we observed isotropic and anisotropic band organization disruption in degenerative cardiomyocytes, widespread edematous areas in myocardial tissue, and significant increases in HPDS scores with IRI.

Caspase-3 is an important enzyme involved in inflammatory processes and apoptosis. Studies investigating IRI in myocardial tissues have reported increased caspase-3 expression in myocardia exposed to oxidative stress and that this results in apoptosis^[17,18]^. Cao et al.^[18]^ showed that the size of the necrotic area, the number of apoptotic cells, and the expression of caspase-3 increased in myocardial tissue exposed to IRI. Yu et al.^[17]^ also showed an increased interstitial fibrosis area, induction of myocardial apoptosis in cardiomyocytes, and a marked increase in caspase-3 activity in their IRI group. IRI was also found to increase caspase-3 activity and apoptotic cardiomyocyte numbers in the present study.

Reactive oxygen species (ROS) produced in normally functioning tissues perform important physiological functions in healthy metabolic processes. However, in case of overproduction during IRI, they result in cell damage through such mechanisms as deoxyribonucleic acid (or DNA) injury and lipid peroxidation in the cell membrane^[13,14]^. MDA, one of the final products of lipid peroxidation, is used as a marker to show damage in tissue caused by oxidative stress^[14,19]^. Studies have shown that IRI causes an increase in MDA levels in various tissues^[2,20]^. Although the number of studies using an aortic clamping model is low, studies focusing on the myocardium have found that IRI causes an increase in MDA in tissue^[14,21]^. Similarly, in the present study, MDA levels increased significantly in myocardial tissue exposed to IRI.

Accumulating FOR plays an important role in cellular damage in tissues and the aging process. The deleterious effects of FOR, the basic cause of oxidative stress, in healthy tissues are kept under control by enzymatic and non-enzymatic balance systems. GSH, catalase (CAT), and superoxide dismutase (SOD) are the most important enzymes in the antioxidant defense system and serve as FOR scavengers. Conditions in which FOR are overproduced, such as IRI, result in the depletion of cellular stores of these endogenous antioxidant enzymes responsible for detoxification^[13,14]^. Liu et al.^[22]^ showed that the amount of SOD, CAT, and GSH in myocardial tissue, in which IRI was induced using the LAD occlusion model, decreased significantly due to consumption. Another study using the same model reported that IRI damage reduced the amount of SOD and GSH in myocardial tissue^[14]^. Consistent with these findings, the present study also determined a significant decrease in GSH levels in myocardial tissue under the effect of IRI.

Studies in recent years have focused on the consumption of fruit and vegetables rich in polyphenols reducing the incidence of cardiovascular diseases. RVT, abundantly present in black grape skin and seeds and with several pharmacological effects, is a polyphenol with powerful antioxidant properties^[4,13,23]^. Studies examining the effects of RVT against IRI in cardiac tissue have considered the local effects of IRI using a LAD occlusion model. In one study, Li et al.^[4]^ determined that RVT reduced caspase-3 expression deriving from IRI and lowered MDA levels in myocardial tissue. Those authors concluded that RVT effectively reduced oxidative stress levels in myocardial tissue and ameliorated IRI. Cheng et al.^[14]^ observed that RVT increased GSH and SOD biosynthesis in myocardial tissue, reduced MDA, and also improved the cardiac functions of the myocardium by reducing the infarct area. Dernek et al.^[13]^ showed that RVT enhanced myocardial healing following ischemia and was effective in reducing arrhythmias and mortality caused by reperfusion. Kazemirad et al.^[23]^ found that RVT reduced MDA levels in ischemic myocardial tissue, enhanced antioxidant capacity, reduced the release of lactate dehydrogenase and creatine kinase, increased heart cell survival by shrinking the infarct area, and increased mechanical performance of the heart by lowering the incidence of arrhythmia. In the present study, RVT caused a decrease in MDA and an increase in GSH levels in myocardial tissue exposed to IRI and led to a decrease in HPDS scores. Additionally, consistent with these findings, we observed decreases in isotropic and anisotropic band disorganization, edematous areas in myocardial tissue, and numbers of degenerative and apoptotic cardiomyocytes with the application of RVT.

### Limitations

To the best of our knowledge, the present study is the first to focus on the potential effects of RVT against IRI developing in the myocardium with clamping of the abdominal aorta as a distant organ in an rAAA model. In addition, there are some limitations to our study. A group exposed to IRI and receiving only DMSO was constituted against the possibility of DMSO, used as the RVT solvent, affecting the results. However, the value of our results can be enhanced if the findings are supported by markers such as other antioxidants and cardiac enzymes. In addition, this research is a pilot study of the effects of RVT as an agent. Our results now need to be supported by pharmacological studies if our data are to be converted into results capable of use in medical treatment.

## CONCLUSION

The findings of the present study indicate that RVT exhibits protective effects against IRI occurring in the myocardium as a distant organ as a result of abdominal aorta clamping. We, therefore, think that RVT may lead to the development of new therapeutic agents for the preservation of myocardial functions and the prevention of multi-organ failure after rAAA surgery.

## Abbreviations, Acronyms & Symbols

CAT: Catalase
CPS: Caspase-3 primary antibody immune positivity score
DMSO: Dimethyl sulfoxide
FOR: Free oxygen radical
GSH: Glutathione
H&E: Hematoxylin and eosin
HPDS: Heart pathological damage score
I/R: Ischemia/reperfusion
IRI: Ischemia-reperfusion injury
LAD: Left anterior descending artery
MDA: Malondialdehyde
MAP: Mean arterial pressure
rAAA: Ruptured abdominal aortic aneurysms
ROS: Reactive oxygen species
RVT: Resveratrol
SOD: Superoxide dismutase
TBA: Thiobarbituric acid
